# Big Epidemiology: The Birth, Life, Death, and Resurgence of Diseases on a Global Timescale

**DOI:** 10.3390/epidemiologia5040047

**Published:** 2024-11-06

**Authors:** Nicola Luigi Bragazzi, Thorsten Lehr

**Affiliations:** 1 Laboratory for Industrial and Applied Mathematics (LIAM), Department of Mathematics and Statistics, York University, Toronto, ON M3J 1P3, Canada; 2 Human Nutrition Unit (HNU), Department of Food and Drugs, University of Parma, 43125 Parma, Italy; 3 Postgraduate School of Public Health, Department of Health Sciences (DISSAL), University of Genoa, 16126 Genoa, Italy; 4 United Nations Educational, Scientific and Cultural Organization (UNESCO), Health Anthropology Biosphere and Healing Systems, University of Genoa, 16126 Genoa, Italy; 5 Department of Clinical Pharmacy, Saarland University, 66123 Saarbrücken, Germany; thorsten.lehr@mx.uni-saarland.de

**Keywords:** big history, epidemiology, epidemiological methods, big data, big epidemiology, biological anthropology, paleopathology, bioarcheology

## Abstract

Big Epidemiology represents an innovative framework that extends the interdisciplinary approach of Big History to understand disease patterns, causes, and effects across human history on a global scale. This comprehensive methodology integrates epidemiology, genetics, environmental science, sociology, history, and data science to address contemporary and future public health challenges through a broad historical and societal lens. The foundational research agenda involves mapping the historical occurrence of diseases and their impact on societies over time, utilizing archeological findings, biological data, and historical records. By analyzing skeletal remains, ancient DNA, and artifacts, researchers can trace the origins and spread of diseases, such as *Yersinia pestis* in the Black Death. Historical documents, including chronicles and medical treatises, provide contextual narratives and quantitative data on past disease outbreaks, societal responses, and disruptions. Modern genetic studies reveal the evolution and migration patterns of pathogens and human adaptations to diseases, offering insights into co-evolutionary dynamics. This integrative approach allows for temporal and spatial mapping of disease patterns, linking them to social upheavals, population changes, and economic transformations. Big Epidemiology also examines the roles of environmental changes and socioeconomic factors in disease emergence and re-emergence, incorporating climate science, urban development, and economic history to inform public health strategies. The framework reviews historical and contemporary policy responses to pandemics, aiming to enhance future global health governance. By addressing ethical, legal, and societal implications, Big Epidemiology seeks to ensure responsible and effective epidemiological research and interventions. This approach aims to profoundly impact how we understand, prevent, and respond to diseases, leveraging historical perspectives to enrich modern scientific inquiry and global public health strategies.

## 1. Introduction

Health conditions and diseases are inherently complex, arising from a myriad of diverse but closely interconnected, interacting, and cascading factors, including biological, environmental, behavioral, and societal influences [1,2,3]. Understanding these determinants and effectively managing diseases necessitates a multidisciplinary approach, involving the collaboration of various fields. These traditionally include basic and translational disciplines, spanning from molecular and cellular biology to genetics, physiology, pathology, physiopathology, and pharmacology, as well as applied ones, like epidemiology, public health, clinical medicine, and clinical public health.

To a lesser extent, the comprehension of diseases has leveraged findings from psychology and human behavior research [4], social sciences [5], and humanities—from literature to history [6,7,8], fine arts [9], geography [10], anthropology [11,12], and even politics and economics [13,14,15], among others. However, despite being relatively overlooked, the integration of these disciplines would allow for a comprehensive analysis and forecasting of disease dynamics, considering not only genetic predispositions but also evolutionary histories, trajectories, environmental exposures, behaviors, and the impact of policies.

Diseases indeed have a history and are deeply intertwined with human history, emerging, evolving, and re-emerging over time [16,17]. As such, a historical lens coupled with an “ecological” and “population” perspective is crucial, recognizing that individual’s health is influenced by a dynamic, complex interplay of different factors, including various diseases and health conditions—a concept known as pathocenosis [18,19,20]. Pathocenosis refers to the coexistence and interaction of different diseases/disease states within a population, shaping the overall health dynamics and epidemiological profile of the community. This concept underscores the importance of considering multiple health conditions and their non-linear interactions rather than focusing on individual diseases and factors in isolation.

Pathocenosis is an example of complexity in health, similar to comorbidity and polypharmacy, where multiple diseases and medications interact, leading to intricate and often unpredictable effects on an individual’s and population’s health.

Recent advancements in genomics, evolutionary biology, and evolutionary medicine have added new layers of complexity to our understanding of disease dynamics [21,22]. Nearly all genetic variants that influence disease risk have human-specific origins, yet the systems they impact trace back to ancient evolutionary events [23]. Human populations exhibit differences in the prevalence of many common and rare genetic diseases due to their diverse environmental, cultural, demographic, and genetic histories. For instance, the genetic architecture of diseases can vary significantly between populations, shaped by a complex web of demographic and historical events [24]. Events such as population bottlenecks [25,26], where a significant reduction in population size due to environmental events or human activity results in a loss of genetic diversity, can lead to unique genetic signatures within a population that influence susceptibility to certain diseases. Introgression [27], the incorporation of genes from one population into another through interbreeding, can also contribute to the genetic diversity and disease risk profiles of different groups. Dramatic historical events, like the transatlantic slave trade, not only forcibly displaced millions of people but also introduced new genetic variants into the Americas, influencing and shaping the genetic makeup [28] and health outcomes of descendant populations [29,30,31,32,33]. Similarly, migrations [34,35], whether voluntary or forced, have led to the mixing of diverse gene pools, bringing together different genetic traits that can affect disease prevalence and response to treatments. Intercontinental explorations and colonization [36] facilitated the spread of pathogens and genetic material across previously isolated populations, further diversifying the genetic landscape or even threatening the health of these populations. Military expeditions and wars [37] have often resulted in the movement of large groups of people, leading to genetic exchanges, the spreading of pathogens, and the introduction of diseases to new regions. However, in some cases, the military has also played a crucial role in combating diseases by facilitating vaccination efforts and supporting public health initiatives [38,39]. Finally, the industrial and post-industrial revolutions [40,41] brought about massive social and environmental changes, including urbanization, globalization, and altered living conditions, which have had long-term effects on human health and genetic variation. These historical events have left lasting imprints on the genetic and biological composition of human populations, influencing not only disease susceptibility but also the effectiveness of medical interventions and public health strategies.

Additionally, evolutionary trade-offs and antagonistic pleiotropy play pivotal roles in disease dynamics. Genes that provide adaptive advantages in certain environments can predispose individuals to diseases in different contexts. For example, alleles that protected against infectious diseases in ancestral environments might increase susceptibility to autoimmune disorders in modern settings [42,43]. Similarly, traits optimized for reproductive success can lead to an increased risk of diseases like cancer or neurodegenerative disorders due to trade-offs in resource allocation and metabolic and other cellular functions [23,44].

Therefore, considering the historical context of human evolution is essential to understanding disease mechanisms [23,44]. The evolutionary history of traits, including ancient adaptations and recent changes, can illuminate why certain individuals and populations are more susceptible to specific diseases. This historical perspective provides valuable insights into how past environmental exposures, evolutionary pressures, and genetic adaptations can have long-lasting and far-reaching implications, influencing current health outcomes.

Understanding pathocenosis, disease ecology, and disease dynamics, and, broadly speaking, the evolutionary context of health conditions and diseases, can provide a more holistic view of global public health, guiding more effective interventions and health policies to improve population health outcomes. By integrating diverse perspectives and expertise, we can gain a comprehensive understanding of disease mechanisms, identify at-risk populations, and develop holistic management strategies that address not only the biological aspects but also the social determinants of health. This collaborative approach is essential for improving health outcomes and ensuring effective, sustainable disease control and prevention.

## 2. The Traditional Epidemiology Paradigm

The traditional epidemiology paradigm is primarily focused on identifying and quantifying the factors that influence the occurrence and distribution of diseases within specific populations [45]. This approach emphasizes the study of the relationships between various risk factors—such as genetic, environmental, and behavioral components—and health outcomes, often through the use of observational studies, such as cohort, case–control, and cross-sectional studies, and randomized controlled trials (RCTs), the objective of which is to identify causative factors and establish associations that can inform public health interventions and policies aimed at disease prevention and control. As such, traditional epidemiology relies heavily on statistical analysis to discern patterns and determine the significance of findings, often operating within a framework that seeks to isolate individual variables and their direct impacts on health outcomes. This paradigm typically involves the formulation of hypotheses based on existing knowledge and testing them through empirical data collection and analysis, with the goal of reducing the incidence of disease and improving overall public health.

The traditional epidemiology paradigm, while foundational in public health, has several limitations and drawbacks: traditional epidemiology often relies on surveillance data, which means it tends to be reactive rather than proactive. This approach typically identifies and responds to disease outbreaks after they have occurred, rather than predicting and preventing them before they start. Moreover, classical epidemiology rarely embraces complexity and operationalizes complex systems thinking [46,47,48,49]. The traditional model tends, indeed, to isolate individual risk factors, such as genetics or specific environmental exposures, without adequately considering the complex interactions between multiple factors. This reductionist approach can miss the broader context in which diseases develop, including cultural practices, and generally fails to capture the non-linear, emergent, and adaptive nature of diseases.

This approach, indeed, relies on the causal inference framework, which is structured to mimic the RCT, a method that, as previously mentioned, is specifically designed to identify the impact of individual factors by isolating them. At its core, this framework presents challenges when applied to the multifaceted nature of public health phenomena and interventions, where the interplay of numerous variables complicates such isolation [47].

## 3. The Big Epidemiology Paradigm

Under these premises, “Big Epidemiology” (Table 1) represents a novel, emerging approach and, more specifically, the conceptual extension of Big History [50,51,52,53,54], which integrates insights from different disciplines, emphasizing the interconnectedness of all events across time and space. Similarly, Big Epidemiology seeks to apply a vast, interdisciplinary approach, in order to understand disease patterns, causes, and effects across human history on a global scale.

This comprehensive framework aims to address present and future global public health challenges by viewing them through a broad historical and societal lens. The foundational research agenda for Big Epidemiology begins with mapping the historical occurrence of diseases and their impact on societies over time. This involves combining historical data (including archeological findings or historical records), biological data (such as genetic and post-genetic data), and clinical data to trace the origins, spread, and societal impacts of diseases.

Traditionally, this has been done with major infections, like plague [55,56,57,58,59,60], smallpox [61], tuberculosis [62,63,64], and influenza [65,66], but the approach extends to noncommunicable diseases as well, like malignancies [67,68], sleep disorders [69,70,71,72,73], or chronic cardiovascular [74,75,76] and neurodegenerative diseases [77,78], adopting multidisciplinary techniques and methodologies that synthesize the diverse sources of information available.

Starting with archeological findings, physical traces of past human populations and their environments can provide crucial insights into historical disease outbreaks. This includes the analysis of skeletal or mummified remains which can reveal signs of diseases, such as lesions indicating arthritis, fractures, syphilis, or tuberculosis [79,80,81]. Additionally, extracting ancient DNA from these remains [82,83] allows for the identification of specific disease biomarkers or pathogens that caused diseases in the past, confirming, for example, the presence of the bacterium *Yersinia pestis*, responsible for the Black Death, in medieval human remains and uncovering its long-term impact on human health [84,85].

Artifacts and ecological data can further complement this picture by indicating the living conditions that may have influenced the spread of diseases, such as evidence of crowding or poor sanitation [86]. Historical records can also play a vital role by providing qualitative (narratives) and quantitative data about disease outbreaks. Chronicles and letters from the past often contain descriptions of symptoms and death tolls, as well as societal responses to these outbreaks. Medical treatises from those periods can offer insights into contemporary understandings of diseases and their treatments, reflecting the medical practices of the time. Furthermore, economic and legal documents, such as records of trade and labor availability, help trace the broader societal disruptions caused by epidemics and other major diseases and provide context for the measures societies took in response to disease outbreaks [87].

Art, sculptures, paintings, and other forms of cultural expression also contribute to our understanding by depicting human experiences during times of disease. These artistic works can reveal how societies perceived and coped with illness, highlighting the emotional and psychological impact of diseases on individuals and communities. For instance, throughout history, artistic representations of infectious diseases and epidemics have documented the physical toll and captured the deep emotional responses to these crises. For example, major catastrophes caused by infectious diseases have pushed artists to depict these events with a stark realism that mirrors the intimate suffering of humanity. Such works, ranging from the death dances and the triumphs of death during the Black Death to the melancholic self-portraits of tuberculosis-stricken artists in the Romantic era, reflect the pervasive fear and the intense emotional turmoil wrought by these epidemics. Artistic depictions of diseases, like the plague, tuberculosis, and even more contemporary afflictions such as AIDS, have served as a powerful means to exorcize the terror associated with these illnesses, offering both a historical record and a means of coping with the collective trauma [88]. Moreover, these works not only capture the historical and emotional landscape of communicable diseases but also serve as a bridge between past and present understandings of noncommunicable disorders. Indeed, they document the presence of various conditions, including congenital malformations and genetic disorders, in historical contexts—often before these conditions were formally recognized by the medical field—thereby providing valuable insights into the evolving perceptions and knowledge of disease throughout human history [89,90,91].

Art can also serve as a historical record of environmental changes, offering valuable insights into phenomena such as air pollution during periods when scientific measurements were not available [92,93]. A recent study [94] suggested that the transition from figurative to impressionistic styles in Turner and Monet’s paintings, characterized by hazier contours and a whiter color palette, may be closely tied to the increased levels of sulfur dioxide (SO_2_) emissions, a key pollutant of that era. The authors employed a quantitative approach using wavelet analysis to measure the contrast in these paintings, finding a significant correlation between decreasing image contrast and rising SO_2_ emissions. This correlation was particularly strong in London, where Turner and Monet painted many of their works. The research revealed that the atmospheric pollution created by industrial activities affected the optical environment, leading to less distinct edges and increased brightness in the air, which these artists captured in their paintings. By doing so, the study introduces a novel methodology for reconstructing historical environmental data through the analysis of artwork, offering a unique way to assess past air quality. By using art as a proxy for environmental conditions, this method enables researchers to extend the timeline of environmental data back to periods before the availability of direct measurements [94,95]. For environmental epidemiology, this approach can provide valuable insights into historical exposure levels to air pollution, thereby helping understand long-term health effects and the evolution of air quality over centuries. This method also bridges the gap between art history and environmental science, illustrating how interdisciplinary approaches can enhance our understanding of environmental changes and their impacts on human health.

Through all these diverse sources, we can piece together a more comprehensive and nuanced picture of how past societies dealt with the challenges posed by widespread disease [96,97].

In recent years, the field of digital humanities [98] has played an increasingly crucial role in advancing our understanding of historical disease outbreaks by digitizing and making accessible a vast array of historical records. Digitization initiatives have transformed fragile manuscripts, medical treatises, personal letters, legal documents, and human remains into searchable digital formats.

Impressive examples of such digitization efforts are digitized datasets on the plague documenting 6929 plague outbreaks in Europe from 1347 to 1900 [58], 7711 outbreaks across Europe and Asia in the same period [99], 5559 outbreaks across Europe and northern Africa2 from 1347 to 1760 [100], and 6656 outbreaks across Europe from 1347 to 1760 [101]. These digital archives offer valuable insights into the spatiotemporal patterns of these historical events, the plague’s transmission routes, its interaction with trade and natural environments, and the possible role of wildlife reservoirs [58,99,100,101].

Another example is “Digitised Diseases” [102,103], an open-access digital archive hosted by the University of Bradford, and a collaboration between the Museum of London Archaeology and the Royal College of Surgeons of England, featuring over 1600 photo-realistic 3D models of pathological human specimens, complete with detailed descriptions, radiographs, CT data, videos, and clinical summaries. This database joins similar archives [104], such as the “Wellcome Osteological Research Database” (WORD) [105], an extensive online repository that contains data from over 35,000 archeologically derived human skeletal remains. A further example is the “Digital Atlas of Ancient Rare Diseases” (DAARD) [106,107,108], a collaborative, publicly accessible database and web-based mapping tool that gathers and displays evidence of various rare diseases identified in skeletons and mummies from around the world, spanning all historical and prehistoric periods. Finally, the “Anthropological and Archaeological Database of Sepultures” (THANADOS) [109,110] and the “Isotopic Database for Bioarcheology” (IsoArcH) [110,111] compile and maintain datasets on anthropologically and archeologically researched burials and on isotopic data, respectively.

This digital revolution enables scholars to employ advanced computational tools, such as text mining, natural language processing (NLP), and data visualization, to analyze large datasets of historical records with unprecedented speed and precision. Moreover, digital archives enable the cross-referencing of disparate sources from different regions and time periods, facilitating more comprehensive and comparative studies of past disease outbreaks.

A concrete example is the Google Books Ngram Viewer [112], which is a tool that enables to investigate historical trends in disease incidence by analyzing the frequency of specific disease-related words in non-scholarly literature. It tokenizes the text into n-grams, which are sequences of one or more words, then counts the occurrences of these n-grams for each year and normalizes the counts by the total number of words published that year, allowing for the visualization of trends. Smoothing techniques can be applied to produce clearer trends, and multiple n-grams can be compared, in various languages and across a range of corpora. For instance, Walker [113] assessed the frequency of scabies, a skin condition caused by *Sarcoptes scabiei mites*, in English literature from 1800 to 2019. While previous research had suggested periodic cycles of 7, 15, and 30 years, employing spectral analysis, a dominant cycle of approximately 32 years could be found. This was confirmed through statistical analysis using periodograms and fast Fourier transform, indicating that word frequency can reflect actual disease incidence. Peaks in scabies-related word usage often correlated with significant historical events like wars, suggesting that societal disruptions may influence disease prevalence. In another study [114], Walker examined the relationship between the frequency of the word “typhus” and historical patterns of epidemic typhus, caused by *Rickettsia prowazekii* and transmitted by body lice. The analysis revealed that usage of the word typhus increased during periods of industrialization and major conflicts, such as World War I and World War II, and declined following public health interventions and the advent of antibiotics. The study found strong correlations between typhus and terms like “conflict” and “warfare”, though less so than expected. The cyclical pattern observed suggested a cycle length of approximately 33 years, aligning closely with the findings in the scabies study. Both studies demonstrate the utility of non-traditional data sources, like Google’s Ngram Viewer, for historical epidemiological research. The analyses relied on LOESS regression to detrend the data and spectral analysis to identify cyclical patterns. Such analyses concluded that word frequency in the literature can serve as a proxy for disease incidence, offering insights especially where historical medical records are scarce.

Similarly, Jurić [115] used Google’s Ngram Viewer to verify the hypothesis that the Russian flu of 1889–1891, which exhibited symptoms like loss of taste and smell, could have been caused by a coronavirus, similar to COVID-19 [116]. The findings revealed a significant increase in the mention of these symptoms during the Russian flu, suggesting a possible similarity in the nature of these pandemics. The study also compared these trends across English, German, and Russian corpora, finding consistent patterns that further support the hypothesis. Even though the hypothesis cannot be definitively confirmed without further historical and medical research, the study demonstrated that Google’s Ngram Viewer is a valuable tool for monitoring trends during pandemics and can offer important historical insights that may inform our understanding of current and future pandemics.

Altogether, these studies highlight how historical text analysis can uncover patterns in disease incidence, correlate with significant societal events, and reflect the impact of public health interventions. Overall, this framework provides a novel perspective on understanding past disease trends and the factors influencing them.

As previously mentioned, genetic data can complement this approach, adding a modern dimension to these analyses by helping trace evolution and historical migration patterns: genomics can enable the reconstruction of the genomes of ancient humans, as well as of ancient pathogens, and comparing them with those of contemporary strains. Also, studying the human genome for markers of disease resistance or susceptibility can shed light on how populations adapted to historical epidemics and disease outbreaks, revealing genetic traits passed down through generations. The “Allen Ancient DNA Resource” (AADR) [117,118], the “Database of ancient human Y haplogroups” (aYChr-DB) [119,120], and the “Ancient mitochondrial DNA database” (AmtDB) [121,122,123] are valuable resources for studying ancient genomes and mitochondrial DNA sequences. Integrating all these sources involves temporal and spatial mapping to pinpoint when and where diseases appeared and how they spread geographically [124,125,126].

An overview of tools, databases, and other resources that can be leveraged in Big Epidemiology studies is shown in Table 2.

## 4. Current Big Epidemiology Projects: An Overview

Currently, there are a few Big Epidemiology projects (Table 3), generally focused on infectious diseases, including Typhoidland [127], an educational and historical initiative that explores the history, science, and global impact of typhoid fever, a disease caused by the bacterium *Salmonella enterica* serotype *Typhi*. Typhoidland is an award-winning international medical humanities project based on the collaboration between University College Dublin and Oxford University, leveraging a unique mix of historical and digital humanities methodologies. It joins a number of other projects that aim to educate the public about the history and control of infectious diseases.

For example, the World Health Organization’s Global Health Histories project [128] documents the history of various diseases and public health challenges, providing a historical context for contemporary health issues. Similarly, the Haiti Lab Cholera Project [129], hosted by the John Hope Franklin Humanities Institute at Duke University, was launched shortly after cholera appeared in Haiti in October of 2010 and explored the history of cholera outbreaks in the Caribbean, providing insight into the spread of the disease in Haiti, where cholera had not previously been present [130,131]. The Smithsonian Institution’s “Outbreak: Epidemics in a Connected World” exhibition [132] delved into the origins and global impact of infectious diseases, highlighting both historical outbreaks and modern challenges such as antibiotic resistance. Additionally, the Spanish Flu Centennial [133] commemorated the 100th anniversary of the 1918 influenza pandemic [134], exploring its global impact and the lessons learned. The Wellcome Collection’s “Contagious Cities” [135] is a multidisciplinary project that examined how urban environments influence the spread of epidemics, combining history, science, and art to explore this relationship. Lastly, the “History of Vaccines” initiative [136] by the College of Physicians of Philadelphia seeks to enhance public awareness of how vaccines function, their development process, and their crucial role in advancing human health. It also addresses the debates surrounding vaccination, as well as the challenges, setbacks, and rare adverse events associated with vaccine use. Many of the historical resources featured on the “History of Vaccines” website come from the extensive collection of rare books, medical journals, manuscripts, and archives housed in the College’s Historical Medical Library. Additionally, the site is accessible in Arabic, Hindi, Spanish, and Urdu.

Altogether, these initiatives contribute to a broader understanding of global public health, disease history, and the importance of preventive measures.

## 5. Big Epidemiology: Integrating Diverse Data

Historical records provide the context necessary to interpret archeological and genetic data, linking disease outbreaks to social upheavals, population declines, or changes in economic systems [23,137]. Genetic studies of both pathogens and human populations can reveal the co-evolutionary dynamics that have shaped the interactions between humans and diseases throughout history [138]. This comprehensive approach enables a deeper understanding of the complex interplay between human societies and diseases over millennia. By learning from the past, we can glean lessons that are crucial for managing health in today’s globalized and rapidly changing world.

Such research will improve our understanding of how diseases have shaped demographic shifts, driven migrations, and catalyzed societal transformations, while also exploring how these factors, in turn, have influenced the spread and impact of diseases.

Another key focus is the co-evolution of pathogens and their human hosts. By utilizing genomic technologies, researchers can study changes in both pathogens and human genetics to understand susceptibility, resistance, and adaptation processes over time. This will inform strategies for managing (re-)emerging pathogens and anticipating future shifts in disease dynamics due to evolutionary changes.

The approach can also be leveraged for analyzing how environmental changes and socioeconomic factors contribute to disease emergence and re-emergence. Integrating data from climate science, urban development, and economic history will assess their roles in disease spread and management, guiding public health planning and interventions that consider long-term and global environmental and economic trends [139].

Global health governance and disease response are also crucial. By reviewing historical and contemporary policy responses to pandemics and disease outbreaks, including institutional roles and international cooperation, the research will enhance future global responses to diseases by understanding what has worked (and what has not worked) in past global public health crises.

Technological and methodological innovations in epidemiology are essential. Innovations in bioinformatics, data integration, and simulation modeling, including the latest achievements and developments in generative artificial intelligence (AI), are needed to handle large-scale, multidisciplinary health data, enabling more precise and predictive epidemiological studies that can better inform public health decisions and interventions [140].

Lastly, the ethical, legal, and social implications of the usage of ancient human remains as well as of large-scale epidemiological studies and interventions should be addressed. Engaging ethicists, legal scholars, and public stakeholders in the development of frameworks that respect individual rights and promote collective health will ensure that Big Epidemiological research and its applications are conducted responsibly and ethically [141,142].

## 6. Big Epidemiology: The Opportunities

Big Epidemiology presents significant opportunities to revolutionize our understanding and management of global health challenges. By integrating insights from diverse fields, this approach can uncover previously hidden connections between diseases and societal factors. For instance, analyzing ancient DNA alongside historical records can provide a detailed picture of how past societies responded to epidemics and disease outbreaks, offering valuable lessons for contemporary public health strategies. This comprehensive perspective enables the identification of long-term trends and patterns in disease spread and evolution, informing more effective prevention and intervention strategies.

Additionally, Big Epidemiology can drive technological and methodological innovations, such as the development of advanced data analytics tools and bioinformatics techniques. These innovations not only enhance research capabilities but also have broader applications in other scientific domains. Moreover, the framework can foster global collaboration, encouraging the sharing of data and expertise across borders, which is crucial for addressing transnational global public health issues.

Ultimately, Big Epidemiology holds the potential to transform public health by providing a deeper, more integrated understanding of how diseases interact with human societies over time, leading to more resilient and adaptive health systems worldwide.

## 7. Big Epidemiology: The Challenges

Big Epidemiology faces numerous challenges (Table 4) in its ambitious goal to integrate diverse disciplines for a comprehensive understanding of disease patterns and impacts.

One significant hurdle is the complexity of merging vast and varied data sources, including archeological findings, genetic data, historical records, and environmental information. Ensuring the accuracy and reliability of historical data, which is often fragmented and contextually diverse, is paramount. Indeed, the uncritical use of such data poses significant risks, including the potential for misinterpretation. For instance, when researchers rely on historical data without thorough analysis or accept it at face value, they may draw incorrect conclusions and create misleading representations of the prevalence and spread of the disease under study. This issue may arise from inherent limitations within the dataset, such as geographic biases (such as the “urban bias”, as data from urban areas tends to be overrepresented due to better-preserved records) and incomplete coverage. A major concern is the lack of critical evaluation of sources, as historical data originates from a variety of sources, each with varying degrees of reliability. Without careful source critique, researchers risk perpetuating errors or biases embedded in the original data. All this can lead to a distorted understanding of historical events. Moreover, the digitization and widespread dissemination of historical datasets can create a false sense of reliability. Once digitized and published in reputable journals, these datasets may be perceived as more accurate or comprehensive than they actually are, leading to their repeated use without adequate scrutiny. This issue is exacerbated when researchers overgeneralize findings from one region or time period to others, resulting in broad but inaccurate inferences. The continuous reuse of outdated or incomplete datasets further obscures the need for new research, fostering the mistaken belief that certain historical topics are fully understood when, in fact, significant gaps remain unexplored. To mitigate these risks, researchers should apply rigorous source criticism, remain vigilant of the limitations and biases within historical data, and strive to verify and expand upon existing data with new research whenever possible [143,144,145].

Moreover, there is considerable debate among historians about whether we will ever be able to accurately identify, from a modern biological perspective, which diseases existed in the past, which diseases were responsible for specific well-known illness episodes, and whether it is advisable to attempt retrospective modern biological diagnoses at all. This is known as the “Cunningham debate” [96,97], arguing against the use of retrospective diagnosis due to the incommensurability of old and new disease concepts, and emphasizing the importance of understanding the social context of past diagnoses rather than imposing modern medical perspectives.

Additionally, not all remains survive equally well over time, and, as such, researchers often work with a skewed sample that may not accurately represent past populations. Cultural factors further complicate this, as historical records or burial practices might disproportionately reflect certain groups, leading to incomplete or biased conclusions. The biased nature of archival records and the uneven preservation of skeletal material are further compounded by the ambiguity of skeletal indicators. Many diseases that leave marks on bones, such as tuberculosis or syphilis, cause similar changes, making it difficult to diagnose specific conditions in ancient populations. Furthermore, some diseases only affect bones after prolonged infection, which means that skeletal evidence may underrepresent the true prevalence of certain pathogens. The process of bone healing and remodeling adds further complexity, as it can obscure whether an individual survived a disease or succumbed to it [146]. The fragility of ancient DNA presents another significant obstacle. Ancient DNA degrades over time, and environmental conditions play a critical role in its preservation. Contamination with modern DNA is also a frequent problem, complicating the analysis of ancient genetic material. Even when ancient DNA is successfully retrieved, it often yields incomplete data, limiting researchers’ ability to reconstruct ancient pathogens or understand their evolution fully. As such, interpreting ancient DNA and correlating it with historical and environmental contexts demands sophisticated technologies and advanced methodologies, which are continually evolving [147,148].

The interdisciplinary nature of Big Epidemiology necessitates collaboration across fields that traditionally operate in silos, requiring mutual understanding and a shared framework for data integration and analysis. Moreover, ethical considerations, such as the privacy of genetic information, the potential misuse of historical data, and the usage of ancient human remains [149], must be carefully navigated. Addressing these challenges involves not only advancing technological and methodological innovations but also fostering an inclusive and ethical research environment. Balancing the need for comprehensive data with respect for individual rights and historical contexts is essential for the responsible advancement of Big Epidemiology. As this field grows, it must continuously adapt to new scientific discoveries and societal needs, ensuring its relevance and impact on global public health strategies.

## 8. Big Epidemiology: The Solutions

Addressing the challenges of Big Epidemiology requires innovative solutions that foster interdisciplinary collaboration and technological advancement. To integrate diverse data sources effectively, the development of standardized protocols and frameworks for data collection, storage, and analysis is crucial [150]. Advanced bioinformatics tools and machine learning algorithms can enhance the accuracy and efficiency of interpreting complex datasets, enabling researchers to uncover patterns and correlations across different disciplines. Establishing interdisciplinary research centers and collaborative platforms can facilitate communication and knowledge sharing among experts from various fields, promoting a holistic approach to understanding disease dynamics. Ethical considerations can be managed by creating robust guidelines that protect individual privacy and ensure the responsible use of historical and genetic data. Public engagement [151,152] and transparent communication [153] are essential to build trust and address societal concerns regarding epidemiological research. Furthermore, fostering international cooperation can help address global health challenges more effectively, leveraging diverse perspectives and resources.

By combining these solutions, Big Epidemiology can overcome its inherent challenges, advancing our understanding of disease patterns and informing public health strategies on a global scale.

## 9. Recent Innovations in Epidemiology and Future Directions

Recent innovations can significantly advance Big Epidemiology, enhancing our understanding of historical and contemporary disease patterns. The integration of big data analytics and machine learning has revolutionized the processing of vast datasets from diverse sources, including historical records, genomic sequences, and environmental data. A concrete example is represented by a novel, NLP-enhanced approach to predicting human life outcomes by using detailed sequences of life events. Savcisens et al. [154] drew upon a comprehensive registry dataset from Denmark, which includes day-to-day records of health, education, occupation, income, address, and working hours for the entire population. They proposed a model called “life2vec” that makes it possible to represent individual life trajectories as sequences, similar to sentences in language, and use these sequences to predict various life outcomes, including early mortality and personality nuances. The life2vec model uses transformer architectures, which are particularly effective at capturing complex patterns in data sequences. By embedding life events into a structured vector space, the model could make accurate predictions about individuals’ futures, significantly outperforming other models like logistic regression and recurrent neural networks. One of the key insights of the study is that the concept space learned by the model was highly structured and meaningful, allowing it to uncover relationships between different life events and predict outcomes with high accuracy. This concept space was also robust, showing consistent results under various conditions. The model’s performance was tested on tasks such as predicting the likelihood of death within four years and predicting personality traits. In both cases, life2vec showed superior performance compared to other baseline models. Other deep learning transformer-based models have been devised that use longitudinal data drawn from electronic health records to predict diseases and their outcomes [155,156,157].

Furthermore, innovations in genomic technologies, particularly next-generation sequencing (NGS), have revolutionized our understanding of genetics and its role in health and disease. NGS allows for rapid and accurate sequencing of entire genomes or specific regions of DNA, making it possible to identify genetic variations that contribute to disease susceptibility. This technology has significantly enhanced our ability to pinpoint specific genes or mutations associated with various diseases, ranging from rare genetic disorders to common conditions like cancer, cardiovascular diseases, and diabetes. In addition to identifying genetic factors in disease susceptibility, NGS has also been instrumental in tracing the evolution of pathogens over time. By sequencing the genomes of viruses, bacteria, and other pathogens, researchers can track how these organisms evolve, spread, and adapt to different environments or hosts. This has been particularly important in understanding the dynamics of outbreaks, such as the COVID-19 pandemic, where genomic surveillance has provided insights into how the virus has mutated and spread across the globe. Furthermore, these genomic advancements have enabled the reconstruction of the evolutionary history of various pathogens, offering clues about their origins, transmission patterns, and how they have co-evolved with human populations. This information is crucial not only for developing effective treatments and vaccines but also for predicting and preventing future outbreaks. Overall, the rapid advancements in genomic technologies, including NGS, have opened up new possibilities for personalized medicine, where treatments and preventive measures can be tailored to an individual’s genetic makeup, and for public health, where understanding the genetic evolution of pathogens can guide global health strategies [158,159]. Specifically concerning ancient genomes, NGS technologies (from shotgun sequencing to targeted enrichment strategies, such as hybridization capture) have significantly increased the amount of DNA sequence data available from extinct organisms, shifting ancient DNA research from a niche field to a central component of evolutionary biology. NGS has enabled the sequencing of entire genomes from extinct species such as the wooly mammoth and Neanderthals, providing new insights into evolutionary processes and human origins. Various library preparation and barcoding strategies have been developed specifically to optimize the sequencing of ancient DNA, balancing efficiency with the need to minimize DNA loss during preparation. In this way, NGS technologies have not only expanded the scope of ancient DNA research but also have the potential to make it a cornerstone of modern genetics [160].

Additionally, advances in geographic information systems and spatial analysis have improved our ability to map disease spread and understand the impact of environmental changes on health [161]. Furthermore, the emerging field of genoeconomics, which studies the genetic influences on economic behavior and outcomes, offers new insights into how genetic factors may intersect with socioeconomic conditions to influence health patterns over time [162,163].

Looking forward, the future of Big Epidemiology lies in further integrating these technologies to develop predictive models that can anticipate disease outbreaks and identify at-risk populations with greater accuracy. Personalized medicine, driven by historical and genetic insights, promises to tailor interventions to individual needs, enhancing treatment efficacy. The increasing emphasis on a One Health approach [164], recognizing the interconnectedness of human, animal, and environmental health, as well as on a Planetary Health approach [165], will guide future research and policy in Big Epidemiology. Collaboration across disciplines and international borders will be essential to addressing global health challenges, ensuring that innovations in Big Epidemiology continue to enrich our understanding and management of diseases on a global scale.

## 10. Big Epidemiology: The Research Agenda and Manifesto

**Figure 1:**
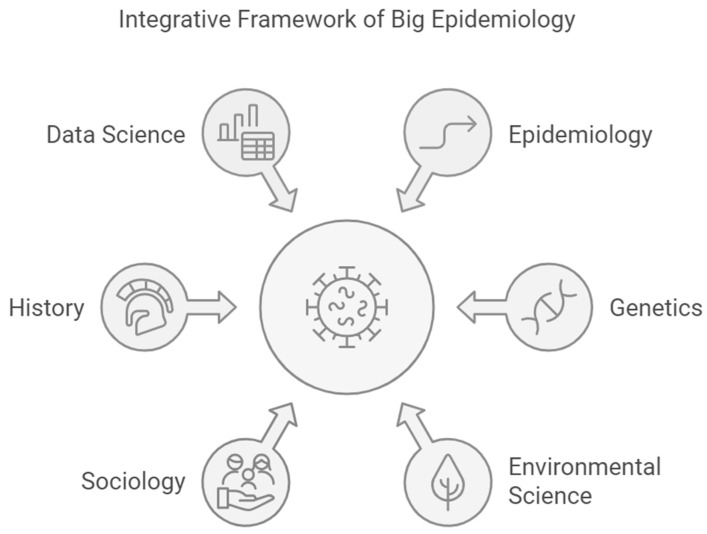
Integrative framework of Big Epidemiology.

The research agenda and manifesto of Big Epidemiology outline a transformative vision for understanding and combating diseases through an interdisciplinary, historical lens. This ambitious framework begins by mapping the historical occurrences and societal impacts of diseases using a vast array of data sources, from archeological findings and genetic data to historical records and environmental studies. The agenda emphasizes the importance of advanced bioinformatics and data integration tools to manage and analyze large, complex datasets. It calls for the creation of interdisciplinary research centers that promote collaboration across fields and foster innovative approaches to studying disease patterns. The manifesto advocates for ethical research practices, ensuring the privacy of genetic information and the responsible use of historical data. It also highlights the need for public engagement and transparent communication to build trust and address societal concerns. By learning from the past, Big Epidemiology aims to inform modern public health strategies, enhance global health governance, and anticipate future disease dynamics. This comprehensive approach seeks to not only understand the co-evolution of pathogens and human populations but also address the socioeconomic and environmental factors contributing to disease emergence and re-emergence, beyond the traditional biomedical model [166,167]. The ultimate goal is to create resilient and adaptive health systems capable of managing current and future public health challenges on a global scale (Figure 1).

There are some features Big Epidemiology shares with some overlapping emerging disciplines (namely, disease ecology/eco-epidemiology [168], social epidemiology [169], critical epidemiology [170,171,172,173], consequentialist epidemiology [174,175,176], evolutionary medicine [177], evolutionary history [178], historical epidemiology [179,180], and the archeology of diseases/paleopathology [181,182]), with distinctive features as well. Table 5 highlights these shared aspects and the distinctions between these fields.

Disease ecology/ecoepidemiology shares with Big Epidemiology a focus on the interaction of multiple diseases within populations, aligning with the holistic view of Big Epidemiology, but it typically does not incorporate historical and evolutionary perspectives.

Big Epidemiology shares with social epidemiology and critical epidemiology a focus on societal determinants of health but expands this view with historical, genetic, and environmental data for a global perspective. Unlike social epidemiology and critical epidemiology, which center on present-day inequalities, Big Epidemiology incorporates long-term historical insights to address disease patterns over time. Consequentialist epidemiology aligns with Big Epidemiology by prioritizing impactful health outcomes, yet it typically emphasizes contemporary interventions. In contrast, Big Epidemiology uses historical data to anticipate future health challenges, aiming for a proactive, globally informed approach.

Evolutionary medicine and Big Epidemiology both emphasize understanding how evolutionary processes influence health and disease patterns, though evolutionary medicine is less focused on integrating historical data and societal impacts.

Evolutionary history provides context on how past human evolution affects current health, contributing to Big Epidemiology’s framework, yet it primarily concentrates on long-term evolutionary changes rather than contemporary disease dynamics.

Historical epidemiology is a field that examines the occurrence, distribution, and determinants of health and disease conditions in historical populations. Big Epidemiology expands its scope and scale, extending these insights into a broader, more integrated framework that aims to address global public health challenges by understanding both historical and contemporary disease patterns.

Lastly, the archeology of diseases/paleopathology offers critical insights into the historical occurrence and spread of diseases, which is integral to Big Epidemiology, but it places less emphasis on current public health strategies and the application of modern technological innovations.

## 11. Policy Implications of Big Epidemiology

The policy implications of Big Epidemiology can be profound, offering a transformative approach to reshaping public health strategies. By integrating historical, genetic, environmental, and societal data, Big Epidemiology can provide a comprehensive understanding of disease dynamics that transcends traditional epidemiological methods. This interdisciplinary framework potentially enables policymakers to anticipate and address future health challenges more effectively by considering long-term trends and co-evolutionary dynamics between pathogens and human populations.

Moreover, the granular insights derived from Big Epidemiology allow for the implementation of precision or population-specific strategies. By identifying genetic, cultural, and environmental factors that influence disease susceptibility in specific populations, public health interventions can be tailored to address the unique needs of different groups, enhancing their effectiveness and efficiency.

The incorporation of historical data and the analysis of past societal responses to epidemics can inform the development of more resilient public health strategies, ensuring that interventions are not only reactive but also proactive and preventive.

Additionally, the ethical, legal, and social dimensions of Big Epidemiology emphasize the need for responsible data use, privacy protection, and the equitable distribution of health resources.

Adopting a historical lens and studying historical health data reveals the critical importance of long-term, data-driven public health planning, emphasizing prevention, equity, and preparedness for (re-)emerging diseases. This highlights the need for policies that integrate socio-economic factors with health interventions to address persistent disparities and ensure a resilient healthcare system, underscoring the value of sustained data collection and global collaboration to effectively manage future public health challenges [183]. As a result, public health policies informed by Big Epidemiology can be anticipated to be more adaptive, inclusive, and globally oriented, ultimately leading to improved health outcomes and more robust global public health governance.

## 12. Conclusions

Understanding and managing health conditions and diseases requires a multidisciplinary approach that integrates genetics, epidemiology, public health, and clinical medicine, as well as social sciences and humanities. The concepts of disease dynamics, disease ecology, and pathocenosis emphasize the importance of considering interactions between multiple health conditions within a population. Recent advancements in genomics and evolutionary biology highlight the role of evolutionary histories and trajectories as well as genetic variations in disease susceptibility.

Considering these premises, the “Big Epidemiology” approach aims to combine historical, genetic, environmental, and societal data to address global public health challenges through an integrated lens. Mapping historical disease occurrences and studying the co-evolution of pathogens and human hosts can provide insights into disease dynamics, guiding future public health strategies. Technological innovations in data analytics, bioinformatics, and AI can enhance research capabilities. To integrate diverse data sources and foster interdisciplinary collaboration, standardized protocols, ethical guidelines, and international cooperation are essential. Public engagement and transparent communication are paramount in building trust and ensuring responsible data use. Big Epidemiology offers significant opportunities to revolutionize our understanding and management of global health issues, creating resilient and adaptive health systems to address current and future public health challenges globally.

In conclusion, through this agenda, Big Epidemiology seeks to profoundly impact how we understand, prevent, and respond to diseases on a global scale, leveraging a historical perspective to enrich and guide modern scientific inquiry and global public health strategies.

## Figures and Tables

**Table 1:** Comparison and contrast between traditional and Big Epidemiology paradigms.

Aspect	Traditional Epidemiology	Big Epidemiology
Focus	Identifies and quantifies factors influencing disease within (sub-)populations	Applies a vast interdisciplinary approach to understand disease patterns on a global scale
Approach	Relies on statistical analysis of observational data (e.g., cohort, case–control, cross-sectional studies) and randomized controlled trials	Integrates historical, biological, and environmental data for comprehensive disease mapping
Scope	Typically focuses on individual risk factors and isolated variables	Emphasizes the complex interplay of multiple factors across time and space
Timescale	Generally constrained to current or near-term data and trends	Considers long-term, historical, and global data for broader temporal analysis
Spatial scale	Typically local or regional, focusing on specific populations or areas	Generally global, considering disease patterns and interactions across multiple regions and environments
Predictive Ability	Generally reactive, focusing on existing data to respond to outbreaks	Aims to be proactive by using historical and genetic data to anticipate future disease dynamics and outbreaks
Data Sources	Primarily uses current and recent epidemiological data	Incorporates archeological, genetic, historical, and environmental data
Interdisciplinary Integration	Limited integration of disciplines, mainly within the public health and clinical fields	Extensive integration across disciplines including history, genetics, sociology, and environmental science
Ethical Considerations	Focuses on data privacy and ethical research within public health	Addresses the broader ethical, legal, and social implications of large-scale data use
Outcomes	Aims to reduce disease incidence and improve public health through targeted interventions	Seeks to inform global public health strategies by understanding the long-term trends and impacts of diseases

**Table 2:** An overview of tools/databases that can be leveraged in Big Epidemiology studies.

Tool/Database	Description	Potential Applications
**Historical record repositories**		
Ancient Infection Digitized Datasets (such as Plague Digitized Datasets)	Digital archives documenting major historical plague outbreaks	Spatio-temporal analysis of plague outbreaks in Europe, northern Africa, and Asia in the last seven centuries
“Digitised Diseases”	Open-access digital archive featuring over 1600 photo-realistic 3D models of pathological human specimens, with detailed descriptions and clinical summaries	Collaboration between the University of Bradford, the Museum of London Archaeology, and the Royal College of Surgeons of England
“Wellcome Osteological Research Database” (WORD)	Extensive online repository containing data from over 35,000 archeologically derived human skeletal remains	It can be used to study historical human skeletal remains and associated pathologies
“Digital Atlas of Ancient Rare Diseases” (DAARD)	Collaborative, publicly accessible database and web-based mapping tool for evidence of rare diseases in skeletons and mummies from various historical periods	It spans all historical and prehistoric periods
THANADOS	A digital archive and tool for studying ancient diseases and osteoarchaeology	Use cases include analysis of skeletal remains and associated diseases
IsoArcH	Database and tools for isotope analysis in archeology and paleoenvironmental studies	Important for understanding diet, migration, and climate interactions in historical populations
**Text mining tools**		
Google Books Ngram Viewer	A tool for analyzing historical trends in disease incidence by examining the frequency of disease-related words in the literature	Study of scabies frequency in English literature (1800–2019) Analysis of historical patterns of typhus epidemics
**Genomic databases**		
Allen Ancient DNA Resource (AADR)	A comprehensive database that provides genetic, genomic, and molecular data on ancient humans	It can be used to trace the evolution and historical evolution and migration patterns It helps study human genome markers for disease resistance or susceptibility
Database of ancient human Y haplogroups (aYChr-DB)	A comprehensive, curated list of all published ancient Y chromosomal haplogroups and annotation	It helps trace human lineage and migration patterns, understand population genetics, and support anthropological and archeological research
Ancient mitochondrial DNA database (AmtDB)	A comprehensive database that provides data on ancient mitochondrial DNA sequences	Understanding genetic diversity and mitochondrial diseases

**Table 3:** An overview of current Big Epidemiology projects.

Project Name	Organization(s)	Description
Typhoidland	Various Institutions (University College Dublin and Oxford University)	An educational and historical project focused on the history, science, and global impact of typhoid fever, with multimedia content and exhibitions
Global Health Histories	World Health Organization (WHO)	It documents the history of various diseases and public health challenges globally, with a focus on historical context and current health issues
The Haiti Lab Cholera Project	The John Hope Franklin Humanities Institute at Duke University	It focuses on the history of cholera in the Caribbean, exploring its major historical outbreaks
Outbreak: Epidemics in a Connected World	The Smithsonian Institution	An exhibition exploring the origins and global impact of infectious diseases, with a focus on historical outbreaks and modern challenges like antibiotic resistance
The Spanish Flu Centennial	Various Institutions	A series of events and exhibitions marking the 100th anniversary of the 1918 influenza pandemic, exploring its global impact and lessons learned
Contagious Cities	The Wellcome Collection	A multidisciplinary exploration of how cities handle epidemics, combining history, science, and art to examine the relationship between urban environments and disease transmission
History of Vaccines	The College of Physicians of Philadelphia	It educates the public on vaccine development, their impact on health and related controversies, drawing on historical resources from the College’s library

**Table 4:** Challenges of Big Epidemiology.

Challenge	Description
Data Integration	Merging vast and varied data sources, including archeological, genetic, historical, and environmental data
Accuracy and Reliability	Ensuring the accuracy and reliability of fragmented and contextually diverse data
Interpretation Complexity	Interpreting ancient DNA and correlating it with historical and environmental contexts requires sophisticated methodologies
Interdisciplinary Collaboration	Facilitating collaboration across traditionally siloed fields, requiring effective communication and shared frameworks
Ethical Considerations	Managing the privacy of genetic information, the potential misuse of historical data, and the usage of ancient human remains
Technological Demands	A continuous need for technological and methodological advancements to handle complex data
Balancing Comprehensive Data with Ethical Responsibility	Maintaining a balance between comprehensive data collection and the respect for individual rights and historical contexts

**Table 5:** Features that Big Epidemiology shares and does not share with potentially overlapping disciplines.

Discipline	Features Shared with Big Epidemiology	Features Not Shared with Big Epidemiology
Disease ecology/ecoepidemiology	Focus on the interaction of multiple diseases within populations, which aligns with Big Epidemiology’s holistic view	Typically does not integrate historical and evolutionary perspectives on disease patterns
Social epidemiology	Emphasis on societal and structural determinants of health, similar to Big Epidemiology’s interdisciplinary approach	Primarily focused on contemporary social inequalities, without extensive historical or global data integration
Critical epidemiology	Addresses power dynamics and social determinants of health, supporting a broader view of disease dynamics	Limited in historical scope and lacks Big Epidemiology’s interdisciplinary, long-term perspective on disease evolution
Consequentialist epidemiology	Focus on maximizing beneficial health outcomes, similar to Big Epidemiology’s proactive approach	Primarily evaluates current health interventions rather than leveraging historical insights for future predictions
Evolutionary medicine	Shares an emphasis on understanding how evolutionary processes impact health and disease, similar to Big Epidemiology’s co-evolutionary focus	Less focused on the integration of historical data and societal impacts
Evolutionary history	Contributes to Big Epidemiology by providing context on how past human evolution affects current health	Primarily concerned with long-term evolutionary changes rather than contemporary disease dynamics
Historical epidemiology	Focus on understanding disease patterns over time using historical data	Lack of integration of modern data science, genetics, and a broader interdisciplinary approach to predict and manage current and future public health challenges on a global scale
Archeology of diseases/paleopathology	Offers insights into the historical occurrence and spread of diseases, which is integral to Big Epidemiology	Less emphasis on current public health strategies and the use of modern technological innovations

## Data Availability

No new, original data was created/generated.
